# Assessing *E. coli* levels in surface soils of informal settlements using boot sock and standard grab methods

**DOI:** 10.1126/sciadv.adq9869

**Published:** 2025-03-14

**Authors:** Lamiya Bata, Rebekah Henry, S. Fiona Barker, John Boyce, Fiona Lynch, Silvia Rosovoa Vilsoni, Autiko Tela, Revoni Vamosi, Ruzka R. Taruc, Andi Zulkifli Agussalim, Maghfira Saifuddaolah, Zainal Handis, David McCarthy, Karin Leder, RISE Consortium

**Affiliations:** ^1^ Department of Civil Engineering, Monash University, Clayton, VIC 3800, Australia.; ^2^ School of Public Health and Preventive Medicine, Monash University, Melbourne, VIC 3004, Australia.; ^3^ Department of Microbiology, Infection Program Biomedicine Discovery Institute, Centre to Impact AMR, Monash University, Clayton, VIC 3800, Australia.; ^4^ School of Public Health and Primary Care, Fiji National University, Tamavua, Suva, Fiji.; ^5^ Revitalising Informal Settlements and their Environments (RISE), Makassar, Indonesia.; ^6^ School of Civil and Environmental Engineering, Queensland University of Technology, Brisbane, QLD 4000, Australia.; ^7^ School of Environmental Sciences, University of Guelph, Ontario, Canada.; ^8^ Victorian Infectious Disease Service, Royal Melbourne Hospital, Melbourne, VIC 3052, Australia.

## Abstract

Rapid urbanization leads to the growth of informal settlements, where inadequate sanitation infrastructure is common, thus promoting environmental contamination and risk of gastrointestinal infection. Soil contamination contributes to the transmission of enteropathogens, but traditional sampling approaches may poorly indicate public health risks due to limited spatial representation. This study compares traditional grab sampling of soil with a boot sock method, a composite technique designed to better reflect human-pathogen interactions. The boot sock method provided more sensitive detection of *E. coli* and lower inter-replicate variation compared to grab samples. Post hoc power analyses indicated that the boot sock technique required fewer samples to achieve adequate spatial representation across a sampling area than grab samples, potentially improving time and cost efficiency in pathogen exposure risk estimation.

## Teaser

Boot sock sampling is an informative and valuable approach to assessing soil fecal contamination in informal settlements.

## INTRODUCTION

Urban informal settlements, home to more than 1 billion people globally, are defined by insecure land tenure for residents (*1*). These communities typically lack access to government regulated trunk infrastructure for sewage, sanitation, and waste disposal, potentially resulting in heavy fecal contamination of the environment. Residents of urban informal settlements, especially young children aged under 5 years, are therefore vulnerable to poor health outcomes (*2*, *3*), including frequent or chronic gastrointestinal infections (*4*–*7*) manifesting as diarrhea, malnutrition, and/or poor physical and cognitive development (*3*, *8*, *9*).

Enteric pathogens may be transmitted in multiple ways, often conceptualized through the F-diagram—food, fluid, fingers, field, and flies (*10*). Until recently, research assessing methods for interrupting these transmission pathways has been mainly directed at improving water and sanitation management, but an increased focus on environmental sources of transmission has now emerged (*11*–*13*). Soil is a natural reservoir for microbial species, including disease-causing pathogens (*14*). While there has been some research on the transmission of helminthic pathogens via ingestion of soil-contaminated produce and groundwater (*15*, *16*), studies on transmission of bacterial pathogens through soil ingestion, whether inadvertent or via geophagia, are limited (*5*, *17*). This transmission route is particularly relevant for very young children because of their crawling and mouthing behaviors. A modeling study in Bangladeshi informal settlements estimated that children under the age of two ingest an average of 198 mg of soil per day (*18*) which, based on *Escherichia coli* concentrations calculated in informal settlement soils, potentially indicates ingestion of more than 2 to 4 log *E. coli* cells daily (*5*, *7*, *19*–*21*).

The traditional gold standard technique for soil monitoring uses spot-based grab samples (*5*, *20*, *22*), often using a sterile spoon or a spatula to “scoop-up” soil [0 to 15 cm in depth (*23*)], or corers to take depth-integrated samples from ~10 to 45 cm below the surface (*24*–*26*). High levels of microbial diversity and spatial variability in soil (*27*) mean that numerous samples are needed to obtain representative information about contamination in the sampling area, thereby introducing high labor and analytic costs. Further, when monitoring large areas such as entire informal settlements, this technique cannot accurately represent the heterogeneity found in microbial species diversity and abundance across different locations (*28*, *29*). In addition, direct soil exposure for young and crawling children is largely to the surface layer of soils (0 to 2 mm) (*5*), which is often only minimally represented by grab samples that capture a greater soil depth. Therefore, more accurate calculation of pathogen abundance in surface soil is needed for prediction of pathogen transmission risks, especially for microorganisms with low infectious doses (*30*).

Surface layer soil sampling techniques, such as boot sock sampling, use polypropylene socks on the sampler’s feet to collect several microsamples while walking on a designated sampling ground (transect) (*31*). This approach, which generates an integrated sample from across a transect, should produce a sample that represents variation in soil microbial diversity along the transect while simultaneously only sampling the upmost surface layer of soil (*32*, *33*). To date, boot sock studies have been predominantly conducted in broiler houses for detection of common poultry fecal pathogens (e.g., *Salmonella* spp., *Campylobacter* spp.), reporting higher sensitivity than fecal spot samples (*33*, *34*) or cecal sampling (*35*). While boot sock sampling has also occasionally been used in other settings (*31*), methodological issues have prevented clear comparison with traditional grab sampling.

In this study, we directly compared a traditional grab sampling technique for measuring fecal indicator bacteria (*E. coli*) in soil with a boot sock approach, including assessment of spatial variability over large areas in both controlled laboratory and field settings.

## RESULTS

### Laboratory-scale comparison with simulated rainfall scenarios

Comparative analysis was conducted between boot sock and standard grab sampling methods to assess the validity of boot sock sampling in measuring *E. coli* in soil environments. The laboratory-based experiment comprised a total of 24 boot sock/20 side-by-side grab samples under four simulated rainfall scenarios. Quantitative analysis was performed on spiked soil samples, with concentrations normalized on the basis of wet sample weights.

**Fig. 1.**
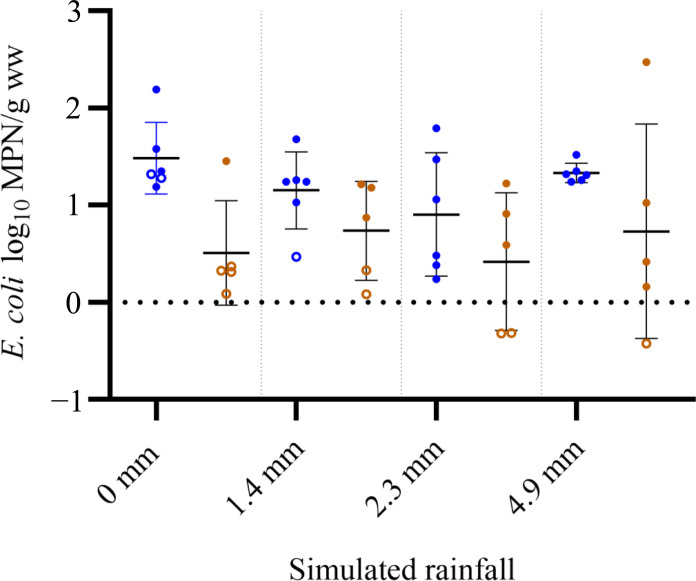
Comparison of *E. coli* concentrations using boot sock and grab sampling techniques under simulated rainfall conditions. Comparison between *E. coli* concentration (log_10_ MPN/g of wet weight of soil) determined via IDEXX Colilert-24 using boot sock (blue symbols) and grab (brown symbols) sampling techniques under four simulated rainfall scenarios in a laboratory transect experiment. Error bars denote means ± SD. Samples with concentrations below detection were calculated by halving the lower limit of detection (shown as empty circles). None of the samples had an *E. coli* concentration above detection limits.

Statistically insignificant but higher overall sensitivity was observed across all four rainfall simulation scenarios using the boot sock approach, with 87% (*n* = 24) of boot sock samples having detectable levels of *E. coli*, compared to 55% (*n* = 20) of grab samples. The largest difference was observed under dry conditions (0-mm simulated rainfall), with 67% of boot sock samples but only 20% of grab samples showing *E. coli* detection (Fig. 1). At simulated rainfall values of 1.4, 2.3, and 4.9 mm, differences of 23, 40, and 20% were observed.

Aggregated results from the inoculated transect indicated that, overall, mean *E. coli* concentrations were higher in boot sock samples compared to grab samples (paired *t* test, *P* = 0.02). In addition, the variability between boot sock replicates was lower than grab replicates (paired *t* test, *P* = 0.02).

### Field-based comparison of sampling methods tested in Fijian informal settlements

**Fig. 2.**
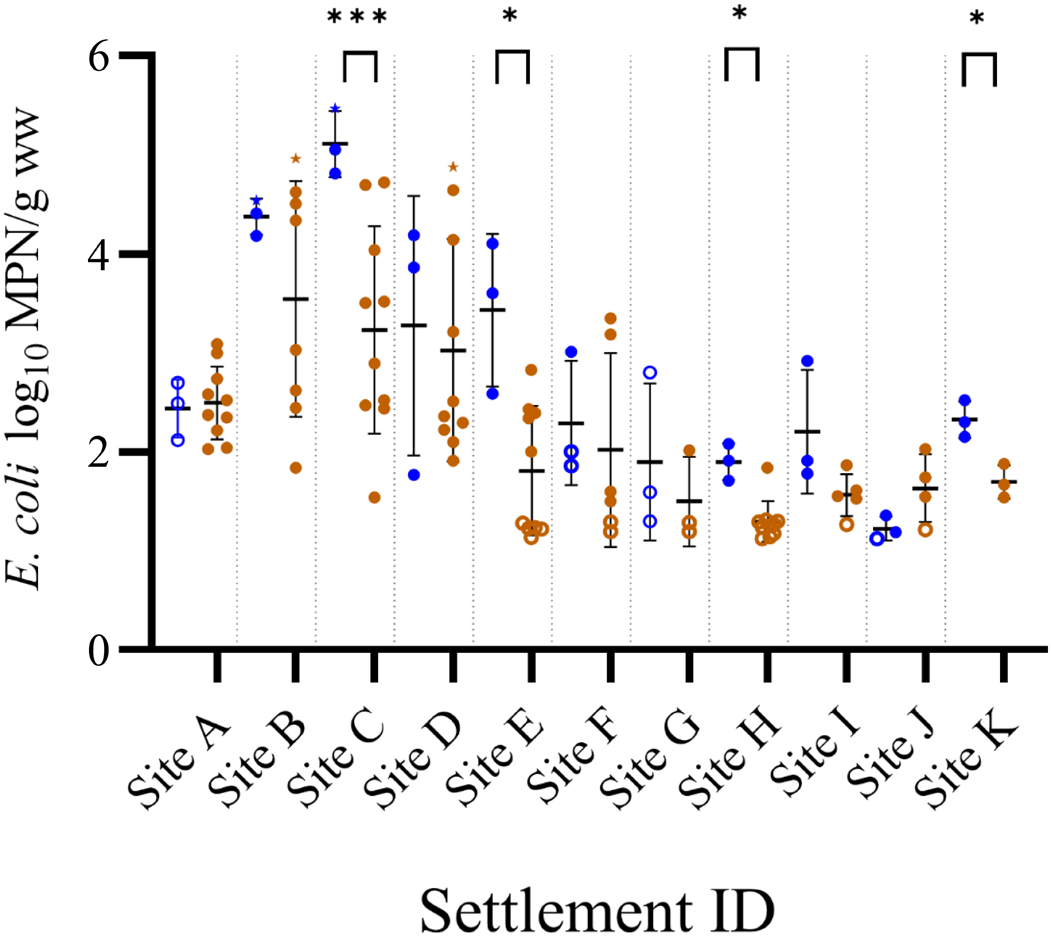
*E. coli* concentrations in informal settlements of Suva, Fiji, using boot sock and grab sampling methods. Comparison between *E. coli* concentrations (Log_10_ MPN/g of wet weight of soil) determined via IDEXX Colilert-24 using boot sock (blue symbols) and grab (brown symbols) sampling techniques from 11 informal settlements in Suva, Fiji. Error bars denote means ± SD. Samples with concentrations below detection were calculated by halving the lower limit of detection and are shown as empty circles. The upper detection limit was used when samples had concentrations above detection (samples indicate with stars).

For field experiments, 33 boot sock/79 grab samples were collected for *E. coli* quantification from 11 informal settlements in Suva, Fiji (Fig. 2). Side-by-side grab and boot sock sampling in these field conditions demonstrated that, for 9 of the 11 sites, boot sock samples had higher mean estimates of transect *E. coli* concentrations compared to grab samples. Overall, this difference was statistically significant (paired *t* test, *P* = 0.01) and driven by data from settlements C, E, H, and K (4 of the 11 sites). In contrast, *E. coli* concentrations were below the method detection limit in boot sock samples from sites A and G, whereas grab samples detected low levels of *E. coli* [1.49 to 2.50 most probable number (MPN)/g wet weight].

The percentage of samples with detectable *E. coli* concentrations was similar for both methods [73% of boot sock samples (*n* = 33); 75% of grab samples (*n* = 79)]. Overall, the mean *E. coli* concentration measured by boot socks was 2.7 log MPN/g wet soil (SD 1.2 log), ranging from 5.5. log MPN/g wet soil (site C) to 1.1 log MPN/g wet soil (site J). The mean *E. coli* estimate using grab samples was 2.3 log MPN/g wet soil (SD 1.1 log), ranging from 5.0 log MPN/g wet soil (site B) to 1.2 log MPN/g wet soil (site H).

In addition to normalizing the *E. coli* concentrations to MPN per gram of wet soil for comparison of grab and boot sock samples, we also explored alternative weighing methods, including an adapted version of the total suspended solids (TSS) measurement method, to provide dry sample weights. This approach aligns with current soil monitoring standards (*23*) and quantitative microbial risk assessment inputs (*36*). The findings from these assessments are provided in the Supplementary Materials (section S2).

### Power analysis

**Fig. 3.**
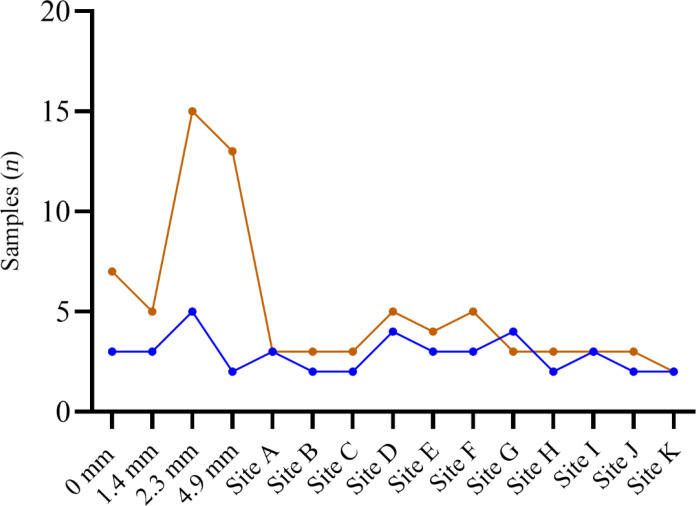
Post hoc power analysis of boot sock and grab sampling techniques. Post hoc power analysis conducted on samples collected using boot sock (blue) and grab (brown) sampling techniques. The *y* axis shows the minimum number of samples necessary to measure transect *E. coli* levels within 95% of the transect mean with a significance level of 0.05 and a power limit of 0.95.

Post hoc power analyses suggested that on average, fewer boot sock than grab samples were required to measure mean *E. coli* levels of the transect (Fig. 3). Sites D and G were exceptions, where a higher number of boot sock samples were required, likely due to greater variability between replicates.

## DISCUSSION

In particular, in settings such as informal settlement environments where fecal waste management is suboptimal, soil can harbor pathogenic microorganisms (*37*) that can infect young children, causing enteric infections and substantial morbidity. Understanding levels of fecal contamination of soil is therefore a crucial element of assessing levels of pathogen exposure risk and predicting public health impacts (*5*). The relationship between fecal indicator organisms and pathogens remains contentious (*38*, *39*), but the use of indicators as an estimate of fecal contamination is still widely used because optimal alternatives are lacking. Here, we have compared *E. coli* detection and quantification using traditional grab sampling versus boot sock sampling in contaminated soil environments, representing the first side-by-side comparison in this context. While we expected similar estimations of *E. coli* from both sampling methods, we found that boot sock sampling consistently yielded higher estimates of *E. coli* in soil samples with lower variation between replicates in both controlled laboratory and field transect experiments. Although the differences between boot sock and grab sample estimates of *E. coli* were not statistically different, this comparison validates that the boot sock method is at least as effective as the current gold standard for soil sampling to estimate fecal contamination.

We found higher sensitivity of the boot sock method, although grab samples sometimes contained up to 10-fold the amount of sampled material. More boot sock samples than grab samples had detectable *E. coli*, and the *E. coli* estimates were higher and showed less variability, indicating that the boot sock method may provide a more time- and cost-efficient sampling method. Fewer samples needed for estimating fecal contamination not only reduces field-worker and laboratory technician time but also reduces requirements for downstream molecular and genomic analyses if performed for detection of individual pathogens. Consequently, there are substantial benefits of progressing this work to evaluate specific pathogen detection by each method, which we intend to test in future work. Our findings support the broader application of the boot sock sampling approach as an important methodological advance for estimating environmental soil contamination in real-world settings, especially when community monitoring of large areas is required.

The higher boot sock *E. coli* concentrations across transects is perhaps explained by the ability of boot socks to exclusively sample the surface layer of soil, which is likely to be most exposed to fecal contaminants through floodwaters, open defecation, and animal scats. In contrast, grab sampling may dilute the *E. coli*–rich surface soil by mixing with less contaminated subsurface soil, leading to lower levels of *E. coli* (*5*, *20*). Given that it is the surface layer of soil that is most likely to enter households on shoes, bare feet, and through open doors and windows (*40*), and more likely to be ingested by children, we consider it optimal for assessing relevant exposure risks to pathogens and consequent likelihood of infection.

Both laboratory and field conditions (with the exception of two Fijian sites) showed that fewer boot sock samples were required for estimating mean *E. coli* levels. This suggests that the efficiency of the boot sock technique holds regardless of environmental moisture and indicates good performance under field conditions even in the face of rainfall variability. However, significant differences in *E. coli* concentrations were observed between boot sock and grab samples at four of the Fijian sites, which could not be attributed to single factors such as tidal influence or soil inputs. Detection rates also varied across sites, with some samples showing concentrations either above or below detection limits for *E. coli*. Future controlled-condition experiments should also include spot contamination, such as the introduction of very high concentrations of *E. coli* in localized areas to represent fecal deposition similar to real world conditions as a way to further test the spatial representativeness of both methods in varying contexts. In addition, future on-site work could help investigate factors influencing microbial pick-up by boot socks, such as why we observed unexplained differences in concentrations between boot sock and grab samples in some study sites. It is possible, for example, that a shallow puddle on the transect containing above-average concentrations of *E. coli* and absorbed by the boot sock elevates concentration estimates compared to grab samples. Factors that could influence findings, such as soil type, road conditions, and varying moisture levels, could be further explored through additional site surveys and sampling.

**Fig. 4.**
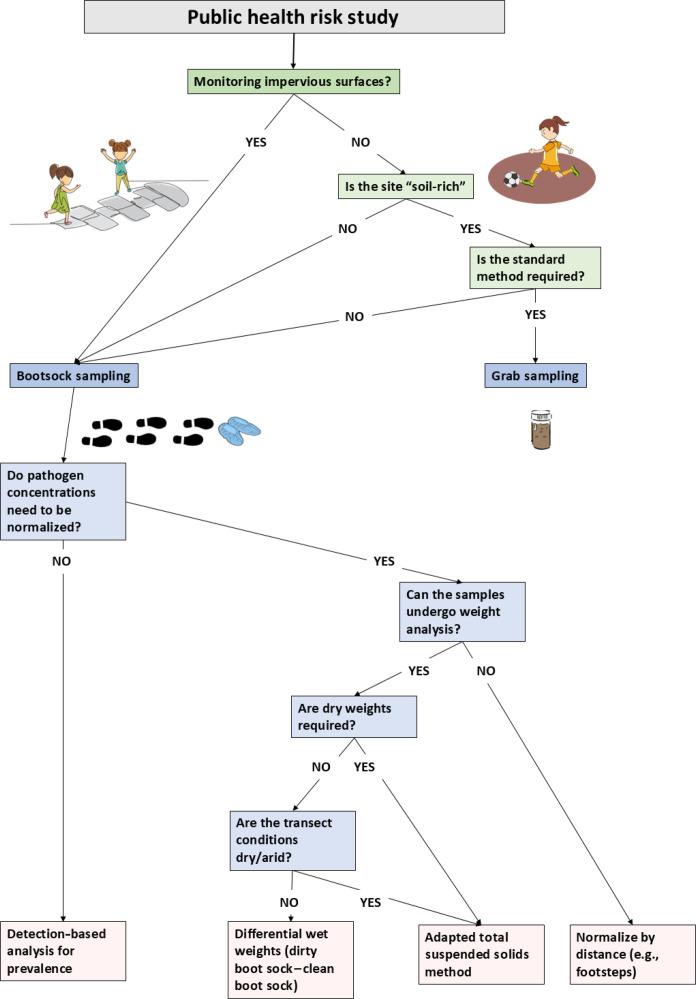
Decision tree for measuring fecal contamination in informal settlement soils. Decision tree showing the best approach for measuring fecal contamination within informal settlement soils. Deeper shades represent decision-making processes at the study level, while lighter squares refer specifically to boot sock sampling. Green squares indicate decision-making steps, while pink squares represent outcomes.

To summarize the knowledge gained from our study and to guide others in developing practical and contextualized methodological approaches, we have developed a decision tree (Fig. 4) and provided sampling recommendations. For instance, in a localized contamination study—such as collecting subsurface soil samples to test for leaky pipes near a toilet—spot-based grab sampling might be optimal, whereas for programs concerned with exposure to pathogens in communities with poor sanitation management, the use of boot sock sampling may be beneficial to achieve broader spatial coverage. Furthermore, we have seen a rise in impervious surfaces globally (*41*). While concrete pathways reduce “soil-rich” environments, these surfaces can still pose a risk of exposure to residents through the dispersal of fine dust particles or direct contact during daily activities. We propose that boot sock sampling enables surface-based sampling, even in areas with limited soil particles. The increased spatial coverage and versatility of the boot sock method can additionally be considered for sampling indoor flooring to assess contamination within the indoor contexts by using distance as a normalization measure.

Despite increasing recognition of the importance of environmental contamination in the spread of childhood gastrointestinal illness, our understanding of soil contamination is hindered by the limitations of current sampling techniques. Traditional grab sampling methods are neither representative of microbial variation in soil, nor do they reflect common human-environment interactions. The boot sock technique offers a composite soil sampling method that may be more efficient than grab sampling methods, but optimization and confirmation of its efficacy in soil environments for microbial detection and pathogen quantification is needed, including optimizing weighing approaches to better normalize across soil samples containing different levels of moisture. Further, the successful application of the TSS method in measuring soil weights showcases the method’s versatility in sampling environmental surfaces in dry or constructed areas. However, irrespective of the normalization approach chosen, it should be understood that possible over- or underestimation of *E. coli* levels may occur and must be considered in a context-specific manner. Our decision tree serves as a useful reference guide in this regard. Our demonstration of increased *E. coli* detection with greater spatial representation at differing soil moisture levels using the boot sock approach indicates the potential of this approach to improve risk assessment of fecal pathogen exposure in contaminated environments. This could be a transformative methodological advance for determining gastrointestinal disease transmission risk, with possible major implications for future public health studies.

## MATERIALS AND METHODS

### Site description

#### Laboratory site

**Fig. 5.**
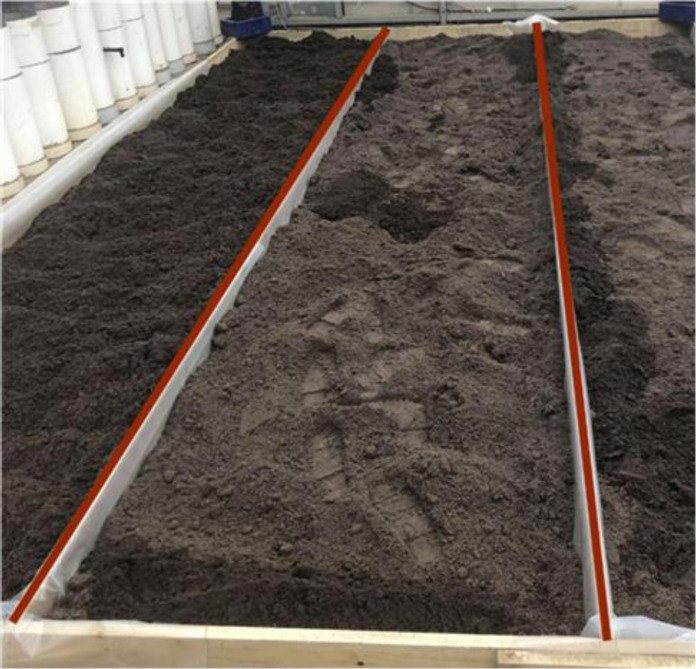
Experimental transect setup for paired boot sock and grab sampling. A 5 m–by–1 m transect used for controlled spiking experiments for the collection of paired soil samples using boot sock and grab methods in laboratory conditions (Monash University, Australia).

Controlled experiments were conducted in a shaded greenhouse at Monash University in Melbourne, Australia. A 5 m–by–1 m constructed walkway or transect was used for spiking experiments (Fig. 5). A plastic tarp was placed inside the frame at ground level, covering all sides of the frame, to prevent water runoff and cross contamination beyond the edge of the frame. The frame was filled by placing lawn mix soil (Daisy Garden Supplies, Australia) onto the plastic tarp and compacting it using a roller. The lawn mix was composed of organic soil mix and sandy loam, blended with organic inputs to facilitate plant growth. Approximately 80 kg of fresh soil was laid out, allowed to dry (~1% soil moisture), and compacted to a depth of approximately 4 cm before the experiment. Three soil subsamples (~5 g each) were collected along the transect to measure baseline concentrations of *E. coli* and soil moisture levels.

The transect soil was inoculated using *E. coli* 332 [strain initially isolated from Troupes Creek, Victoria (*42*)]. Initial concentration of the pure culture was determined using the US Environmental Protection Agency centrifugation method (*43*) and Idexx Colilert-24 (*44*, *45*). Inoculation conditions are outlined in Table 1. Briefly, a 19.9-ml aliquot of pure culture was mixed with 1.8 liter of potable water, and was used to inoculate the transect soil to a final concentration of ≥103.4 MPN/g dry weight of soil. The inoculum was evenly applied to dry soil, across the length of the transect using a watering can. Following inoculation, the soil was irrigated to simulate conditions of average rainfall in Melbourne, Australia and Suva, Fiji (three irrigation events; Table 1) to enable assessment of the impact of soil moisture on soil sampling practices.

**Table 1.** Experimental conditions for boot sock and grab samples collected from the controlled-laboratory transect.

Time (hours)	Experiment description	Transect distance (meter)	Simulated rainfall (millimeter)	Volume (liter)	Boot sock samples (*n*)*	Grab samples (*n*)
0	Inoculation	N/A	N/A	1.82 (used for inoculation)	0	3 (pre-spike)
22	Sampling under dry conditions, no rainfall simulated	50	0	0	6	5
23	Sampling conducted under simulated daily rainfall during Melbourne’s dry season† (*50*)	50	1.40	7	6	5
47	Sampling conducted under simulated daily rainfall during Melbourne’s wet season† (*50*)	50	2.3	11.45	6	5
48	Sampling conducted under simulated daily rainfall during Fiji’s dry season‡ (*51*)	50	4.90	13	6	5

*A total of two separate boot sock samples were collected per walk for a total of six replicates (*n*) over three separate transect walks

^†^Average daily rainfall for Melbourne, Australia.

^‡^Average daily rainfall for Suva, Fiji

#### Field study in Fiji settlements

Eleven RISE informal settlements in Suva, Fiji (18.1416°S, 178,4419°E) were chosen for comparative field sampling between 28 August 28th and 01 September 2018. Soil sampling was conducted along a single transect at each of the 11 sites. Transects were chosen within each community based on sanitary surveys identifying areas of heavy local foot traffic and frequent visits by children. These transects varied in distance (25 to 105 m) and composition (soil, sand, gravel, and concrete). To ensure the privacy and confidentiality of the communities involved, the specific locations of the transect sites have been deidentified. However, transect descriptions, along with blinded identifiers for each community, are provided in Table 2.

**Table 2.** Transect descriptions from 11 RISE informal settlements in Suva, Fiji. Daily rainfall for the 24 hours before sampling was 0 mm in all sampling locations except in H, I, and J where it was 1 mm (climate data accessed with permission from Fiji Meteorological Service, March 2020).

Settlement ID	Transect description	Transect distance (meter)	Boot sock samples (*n*)	Grab samples (*n*)
A	Compact dirt path, main road, exposed	50	3	10
B	Rock path, experiences tidal flooding	30	3	8
C	Dirt, plant debris, shaded	27	3	10
D	Main road, strategic rock placement, exposed	55	3	10
E	Dirt path, site experiences tidal flooding	40	3	10
F	Compact dirt path, main road, exposed	105	3	6
G	Compact dirt path, main road, exposed	57	3	3
H	Sea-facing sport oval, loose soil	65	3	10
I	A lawn outside the community church	61	3	5
J	Compact dirt path, main road, exposed	22	3	4
K	Patch of grass (play area), dirt path	60	3	3

### Soil grab sampling—Laboratory and field

**Fig. 6.**
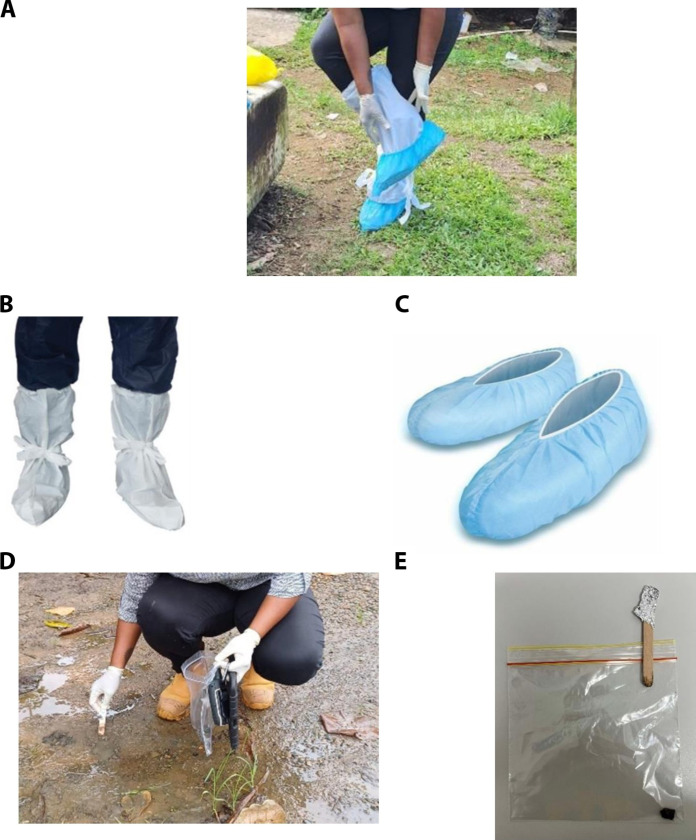
Field sampling for the collection of boot sock and grab samples. In the field, a sampler (**A**) prepares to collect a boot sock sample, wearing overshoes (**B**) and boot socks (**C**). The sampler (**D**) collects a grab sample using a disposable plastic bag and a wooden Popsicle stick (**E**).

The soil grab sampling method (AS4482.1) (*23*) was used to collect 3 to 10 grab samples across the length of the laboratory and field transects (Fig. 6D). Briefly, ~5 g of soil was collected from ~2 cm^2^ area using sterile wooden popsickle sticks (Fig. 6E) every 1 to 10 m along the transect. The samples were placed into food grade plastic bags (Fig. 6E) and transported on ice for processing within 4 hours of collection.

### Boot sock sampling—Field

Side-by-side triplicate boot sock samples were also collected (*n* = 6 boot socks). Before sampling, the weight of each boot sock was recorded to two decimal places and the boot socks were stored in separate disposable plastic bags. Samplers wore gumboots for boot sock sampling to standardize tread placement. Upon reaching the sampling site, the samplers wearing fresh gloves removed the plastic overshoes (Pro-Val 50506; Fig. 6B) from the sampling kit and placed them over their gumboots (Fig. 6A). This plastic layer prevented cross contamination between the sampler’s gumboots and the sampling boot sock. The boot socks (Pro-Val PPSHOE, Fig. 6C) were then placed over the plastic overshoe. As compared to grab sampling, the boot socks approach can be considered surface specific. However, the environmental conditions affecting soil structure can influence the depth of soil profile obtained. Overshoes and boot socks were changed between each boot sock replicate. To confirm the integrity of the boot sock sample, water was poured into the plastic overshoes (after the transect walk) to test for tears and ensure that no contamination had occurred as a result of contact with material from the sampler’s gumboot. This process was repeated to collect six (3 walks by 2 feet) replicate boot sock samples for each transect. One boot sock from each pair was randomly chosen for *E. coli* quantification. A clean weighed boot sock, carried to the field alongside other equipment, was used as a control to test for field contamination.

### Boot sock sampling—Laboratory

Laboratory transect samples were collected as described above but with two modifications. Six replicate boot sock samples were collected for each “rainfall” event by walking back and forth along the transect to cover a total of 50 m. A total of 24 (4 conditions by 3 walks by 2 feet) separate boot sock samples were collected over 2 days of experimentation. All 24 boot socks were processed for *E. coli* quantification. Soil grab samples and boot sock samples were collected concurrently (same time) in each location.

### Sample processing for *E. coli* analysis

Boot sock sample wet weights were determined by difference [post sampling boot sock mass (g) – presampling boot sock mass (g)]. Weights were recorded to two decimal places. For field studies, one boot sock was randomly selected from each pair for *E. coli* analysis. For laboratory-based studies, both left and right boot socks were processed, to expediate the sample collection process. To make all boot sock samples usable in the 2018 field study, weight adjustments were performed by determining the average soil amount collected per meter walked for boot socks showing negative differential weights (see table S1).

The boot sock was then placed in a stomacher bag (Stomach 400 Classic Bags) together with 50 ml of 0.05% phosphate-buffered saline–Tween 80. Each boot sock was stomached at 230 rpm for 1 min (*33*), and the resulting aqueous supernatant was processed for *E. coli* quantification. Grab samples were processed by subsampling 1 to 2 g of soil from each grab sample, weighing to two decimal places to obtain wet weight, and processing for *E. coli* quantification using the same stomaching method as described for boot sock samples (*46*).

### Determination of *E. coli* concentration

The supernatant from either boot sock or grab samples was used for *E. coli* quantification using the IDEXX Colilert-24 system (*44*), with results reported as *E. coli* concentration per gram of wet weight of starting material (MPN/g wet weight). Samples outside of detection limits are reported as equal to the upper detection limit or half of the minimum detection limit (*47*). All concentrations were log transformed before analysis.

### Statistical analysis

Statistical software R-studio (version 4.2.1) and GraphPad Prism (version 9) were used to analyze data in this study. Replicate boot sock and grab samples were used to estimate mean soil contamination across each transect. Normality testing was conducted using the Shapiro-Wilk and Kolmogorov-Smirnov test (*P* < 0.05) to establish the Gaussian distribution of the boot sock and grab sample datasets (*48*, *49*). For each transect, Welch’s *T* test and the Mann-Whitney test were conducted, on the basis of their ascertained normality, to compare estimated mean *E. coli* levels using the two sampling techniques. Nonparametric analysis of variance (ANOVA) (Kruskal-Wallis test; *P* < 0.05) was used to compare *E. coli* levels measured under different soil moisture levels (laboratory samples only).

Post hoc power analyses were conducted on *E. coli* concentrations to determine the minimum number of samples necessary to achieve 95% confidence in measuring mean *E. coli* concentrations for each transect and for each sampling method. The Microsoft Excel TDIST() function with a significance level of 0.05 and a power limit of 0.9 was used to determine *N* (number of samples) needed to ensure mean concentrations fell within 95% of the transect mean.

Weight optimization analyses used aggregated field data collected between January 2022 and December 2023. Statistical analyses were conducted on differential wet and TSS dry weights using nonparametric Wilcoxon and paired *t* tests to compare the methods.

## Supplementary Materials

### This PDF file includes:

Sections S1 and S2 Table S1 Figs. S1 and S2 List of RISE Consortium members
