# Supplementary material for: Assessing E. coli levels in surface soils of informal settlements using boot sock and standard grab methods
Source: Sci Adv. 2025 Mar 14;11(11):eadq9869. doi: 10.1126/sciadv.adq9869 (PMC11908490; doi:10.1126/sciadv.adq9869)
Supplement: Supplementary file 1 — Sections S1 and S2 Table S1 Figs. S1 and S2 List of RISE Consortium members [file sciadv.adq9869_sm.pdf]

Science Advances
AAAS

Supplementary Materials for

Assessing *E. coli* levels in surface soils of informal settlements using boot sock and standard grab methods

Lamiya Bata *et al.*

Corresponding author: Karin Leder, karin.leder@monash.edu; David McCarthy, david.mccarthy@qut.edu.au

Sci. Adv. **11**, eadq9869 (2025)
DOI: 10.1126/sciadv.adq9869

This PDF file includes:

Sections S1 and S2
Table S1
Figs. S1 and S2
List of RISE Consortium members

S1. Weight adjustments for field boot sock samples used for *E. coli* quantification

15% of boot sock differential weights from the 2018 field study were negative, and thus, unusable for the purpose of normalizing *E. coli* concentrations. These errors were attributed to the overall low sensitivity of the method in measuring small differences in sample weights due to low levels of soil pick up and human error. Thus, weight adjustments were made to boot sock sample weights that also underwent *E. coli* analysis. These weight adjustments relied on the assumed relationship between distance sampled and soil collected by replicate boot socks collected from the same transect. This assumed relationship was leveraged to extrapolate sample weights for boot socks and is shown in Table S1.

Table S1.

Sample weights from boot sock samples collected in Suva, Fiji in 2018. Negative differential weights were observed in five individual boot sock samples, shown in bolded text. These samples underwent weight adjustments (Adjusted Soil ww (g)) for microbial quantification via assuming the mass of soil (Soil ww (g)) collected based on the distance walked (Distance (m)), extrapolated from boot sock replicates from the same transects. *E. coli* concentrations for each boot sock replicate, normalized by wet weight, are provided, with concentrations italicized for values below the detection limit.

**Site**	**Replicate**	**Ziploc + BS (g) (Pre-sampling)**	**Ziploc + BS (g) (Post-sampling)**	**Soil ww (g)**	**Distance (m)**	**Soil ww g/m**	**Average Soil ww g/m**	**Adjusted Soil ww (g)**	***E. coli* MPN/g ww**
**0 mm rainfall**	1	8.06	8.18	0.12	n/a	n/a	n/a	0.12	*20.83*
	2	8.1	8.2	0.1	n/a	n/a	n/a	0.1	155.00
	3	8.09	8.22	0.13	n/a	n/a	n/a	0.13	*19.23*
	4	8.04	8.2	0.16	n/a	n/a	n/a	0.16	15.63
	5	8.1	8.25	0.15	n/a	n/a	n/a	0.15	38.27
	6	8.16	8.31	0.15	n/a	n/a	n/a	0.15	22.22
**1.4 mm rainfall**	1	7.99	10.09	2.1	n/a	n/a	n/a	2.1	48.10
	2	7.9	10.71	2.81	n/a	n/a	n/a	2.81	17.26
	3	8.07	9.52	1.45	n/a	n/a	n/a	1.45	10.69
	4	7.99	9.73	1.74	n/a	n/a	n/a	1.74	18.39
	5	8.09	8.93	0.84	n/a	n/a	n/a	0.84	2.98
	6	7.96	8.85	0.89	n/a	n/a	n/a	0.89	17.42
**2.3 mm rainfall**	1	8.03	15.37	7.34	n/a	n/a	n/a	7.34	1.74
	2	8.06	14.9	6.84	n/a	n/a	n/a	6.84	2.38
	3	8.09	11.95	3.86	n/a	n/a	n/a	3.86	29.61
	4	8.05	13.88	5.83	n/a	n/a	n/a	5.83	3.00
	5	8	11.06	3.06	n/a	n/a	n/a	3.06	61.07
	6	8	11.41	3.41	n/a	n/a	n/a	3.41	11.61
**4.9 mm rainfall**	1	7.88	19.29	11.41	n/a	n/a	n/a	11.41	18.04
	2	8.04	17.54	9.5	n/a	n/a	n/a	9.5	22.59
	3	8.03	15.29	7.26	n/a	n/a	n/a	7.26	21.13
	4	8.08	15.36	7.28	n/a	n/a	n/a	7.28	17.33
	5	8.08	13.48	5.4	n/a	n/a	n/a	5.4	20.41
	6	7.94	12.09	4.15	n/a	n/a	n/a	4.15	33.45
**Site A**	1	9.95	10.14	0.19	50	0.004		0.19	*131.58*
	2	9.91	9.99	0.08	50	0.002		0.08	*312.50*
	3	10.26	10.31	0.05	50	0.001	0.002	0.05	*500.00*
**Site B**	1	9.73	9.58	**-0.15**	30	-0.005		*3.32*	25613.22
	2	9.13	12.1	2.97	30	0.099	0.111	2.97	35067.34

	3	9.13	12.8	3.67	30	0.122		3.67	15241.14
**Site C**	1	9.63	10.39	0.76	27	0.028		0.76	112486.84
	2	10.16	10.86	0.7	27	0.026		0.70	64328.57
	3	10.78	11.13	0.35	27	0.013	0.022	0.35	297571.43
**Site D**	1	10.24	10.69	0.45	55	0.008		0.45	7288.89
	2	9.55	10.4	0.85	55	0.015		0.85	58.82
	3	10.31	10.85	0.54	55	0.010	0.011	0.54	15620.37
**Site E**	1	9.89	10.02	0.13	40	0.003		0.13	384.62
	2	9.74	9.82	0.08	41	0.002		0.08	3937.50
	3	10.4	10.27	**-0.13**	41	-0.003	0.003	*0.11*	12473.62
**Site F**	1	10.74	10.94	0.2	105	0.002		0.20	1025.00
	2	9.65	9.6	**-0.05**	105	-0.0004		*0.23*	*71.88*
	3	9.78	10.03	0.25	105	0.002	0.002	0.25	*100.00*
**Site G**	1	10.47	10.51	0.04	57	0.0007		0.04	*625.00*
	2	10.34	9.67	**-0.67**	57	-0.012		*0.65*	*38.46*
	3	9.26	10.52	1.26	57	0.022	0.011	1.26	*19.84*
**Site H**	1	9.92	11.89	1.97	48.75	0.040		1.97	50.76
	2	10.4	11.02	0.62	63.75	0.010		0.62	80.65
	3	9.57	11.73	2.16	68.25	0.032	0.027	2.16	120.37
**Site I**	1	9.95	10.07	0.12	61.5	0.002		0.12	833.33
	2	9.12	9.95	0.83	63	0.013		0.83	60.24
	3	10.34	10.3	**-0.04**	60	-0.0006	0.008	*0.45*	81.43
**Site J**	1	10.08	12.25	2.17	22	0.099		2.17	23.04
	2	9.01	12.27	3.26	22	0.148		3.26	15.34
	3	10.47	12.35	1.88	22	0.085	0.111	1.88	*13.30*
**Site K**	1	9.51	10.21	0.7	58	0.012		0.70	142.86
	2	9.77	10.27	0.5	60	0.008		0.50	200.00
	3	9.88	10.33	0.45	60	0.008	0.009	0.45	333.33

S2. Boot sock– Total Suspended Solids (TSS) evaluation

Boot sock sample collection was undertaken as described above for field sampling, in order to evaluate the potential to use TSS to determine dry weight. Here, between 1-5 transects were sampled per settlement per campaign biannually across June 2022-Dec 2023. For this evaluation, samples were but were taken from within twelve communities in Suva, Fiji (18.1416ºS, 178,4419ºE), eleven communities in Makassar, Indonesia (5.1616° S, 119.4359° E), and from a third field study location at the Jock Marshall Reserve at Monash University Clayton Campus, Australia (37.9095ºS, 145.1397ºE). Boot sock samples at the Jock

Marshall Reserve were collected in triplicate (n=6 boot socks) by walking three different transect lengths: 10, 35, and 100 footsteps to mimic transect lengths found within informal settlements (Fig. S1.).

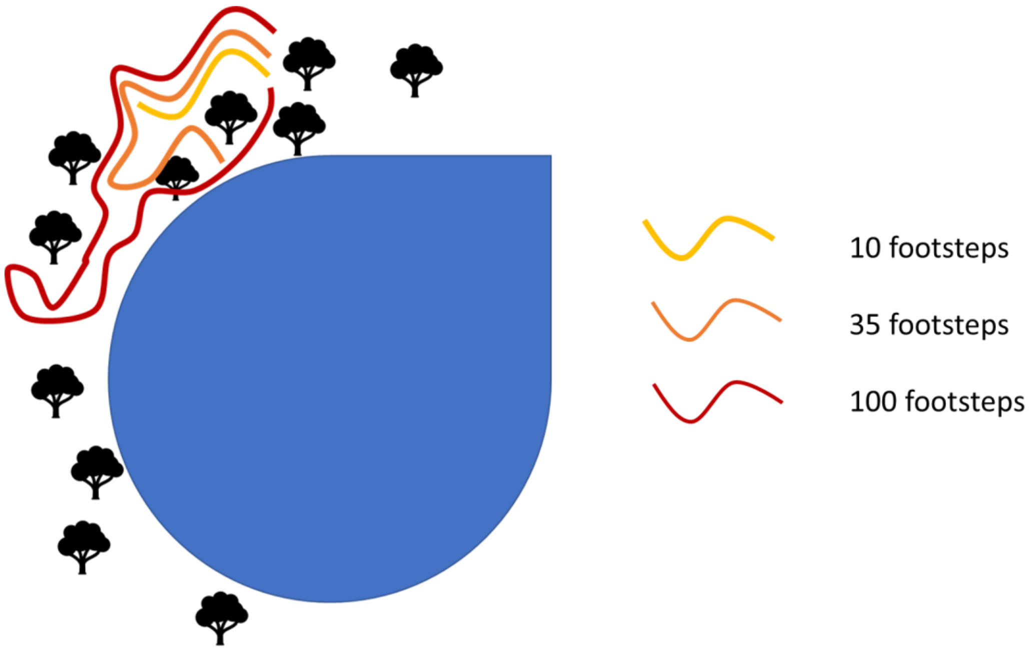

Fig. S1. Walking track for field experiment at Jock Marshall Reserve, Melbourne. A pictorial depiction of the walking track used for the field experiment conducted at the Jock Marshall Reserve. Boot sock samples were collected by walking along the selected transect to cover 10, 35, and 100 footsteps, shown as yellow, orange, and red lines respectively.

Methods – Measurement of TSS from boot sock samples

Obtaining dry weight for normalization of boot sock *E. coli* levels was impeded by the low mass of the sample and the low mass of the boot sock itself. Additional field experiments were therefore performed on boot sock sample supernatants to evaluate calculation of dry sample weight using the TSS method, whereby the dry weight of sample released from the boot sock could be determined following the stomacher processing step. TSS is a standard technique used for water testing and was hypothesised to have improved sensitivity due to concentrating the sample mass on a filter for drying (52).

Each boot sock was weighed before and after sampling to calculate differential **wet** weights. Each boot sock was stomached twice, with independent aliquots of 50 mL 0.05% PBS-Tween 80 buffer to increase soil removal from the boot sock. The supernatant was decanted after each stomaching procedure into separate 50 mL tubes. The total volume of the sample solution (tubes 1 and 2) was combined and processed for the TSS measurement (ASTM D5907) (52). Briefly, a clean aluminium tray containing a 1.5-micron filter was weighed to two-decimal places. The filter was removed from the aluminium tray and placed on a sterile cup holder and securely attached to a filtration manifold. The total sample solution was filtered and the filter gently removed from the manifold and returned to its original tray. The contents of the tray were then dried at 105° C for 24 hours. Once dried, the tray and dirty filter were weighed together to two-decimal places. The TSS content of each sample was calculated using the following formula:

TSS (g) = 100 * (Tray and dirty filter g – Tray and clean filter g) / (Filtration volume mL)

To serve as a negative control, a separate sample consisting of 100 mL of 0.05% PBS-Tween 80 was processed using the TSS method to account for any background weight attributed to the PBS-Tween solution itself.
Weight optimisation analyses used aggregated field data collected between January 2022 and December 2023. Statistical analyses were conducted on differential wet and TSS dry weights using non-parametric Wilcoxon and paired t-tests to compare the methods.

Results - Optimizing boot sock sample weight measurements using TSS
Several factors challenged the normalization of boot sock *E. coli* MPN to soil mass. In the case of wet weight normalization, these were primarily based on the low sample mass and low mass of the boot sock itself. Normalization on the basis of dry weight, in line with the majority of soil-pathogen literature, was unsuitable for the boot sock method due to oven drying and the possibility of transfer-based sample loss. In order to address these issues, and to present data as dry weight normalized, a TSS based approach to soil recovery and drying was evaluated.

Boot sock samples were collected from Jock Marshall Reserve, Australia (n=9), Fiji (n=156), and Indonesia (n=148). As may be expected, the dry weight of soil recovered from boot socks using the TSS approach was consistently and significantly lower than wet weight ($p<0.01$; Fig. S2). This indicated the important contribution of moisture in the soil samples obtained from the boot sock method, supporting the need for a dry weight approach. The TSS approach improved the recovery of meaningful mass data (>0 grams) from the boot sock samples in Makassar, Indonesia (8.8% improvement) compared to (wet) differential weighing. However, a 5.8% decrease in meaningful sample mass was observed for boot sock samples in Suva, Fiji when TSS weights were used over wet weights. Meaningful mass data was obtained from all samples collected from the Jock Marshall Reserve, using both wet weight and TSS methods.

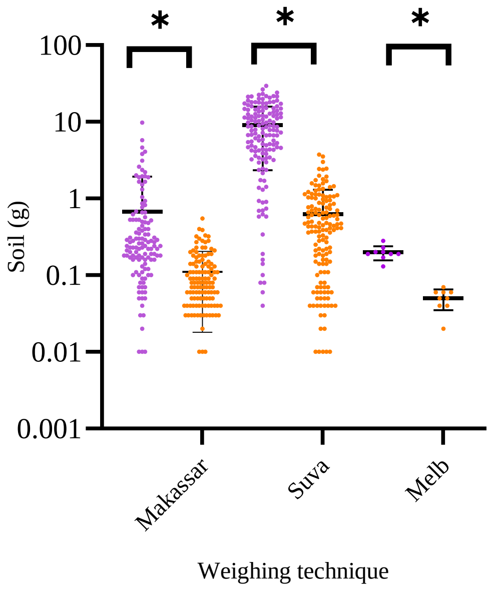

Fig. S2. Comparison of differential wet weights and TSS in soil collected using boot sock sampling.
Comparison between differential wet weights (purple symbols) and TSS dry weights (orange symbols) in the measurement of soil picked up by boot sock samples in Makassar (n=123),

Melbourne (n=9) and Suva (n=147). Only positive weights were used in this analysis. Error bars represent Mean±SD, statistical differences are denoted (Wilcoxon and paired t-test, 0<0.01, *, p<0.001, **). Only meaningful data (positive mass) are presented. Samples excluded totalled: 1 (TSS, Makassar); 14 (ww, Makassar); and 9 (TSS, Fiji).

Discussion – Application of TSS for boot sock samples

A limitation of our initial field boot sock sampling study was that we could not conduct moisture content testing on the boot sock sample itself. This was primarily due to its preservation and transportation in a Ziploc bag. Removal from the bag for weighing would result in unnecessary handling and sample loss and the Ziploc bag itself could not withstand oven temperatures. These factors led to us normalizing *E. coli* results in MPN/g of wet weight of soil when comparing grab and boot sock samples (**Error! Reference source not found.** and **Error! Reference source not found.**). It should be noted that this does differ from conventional soil reporting practices, but likely provides a more realistic measurement of the material collected by the boot sock.

The wet (differential) weight approach to normalization also presented challenges due to the presence of the boot sock itself during the weighing procedure and the low mass of adhered soil, particularly from dry transects. These factors meant that it was challenging to obtain meaningful mass data across a whole dataset. Thus, a TSS-based approached was evaluated for its potential to increase the proportion of meaningful mass data and to provide normalization of *E. coli* MPN on the basis of dry weight. Field experiments demonstrated significant differences between differential wet weights and TSS dry weights obtained from the same boot sock. Differential wet weights were consistently higher than paired TSS dry weights due to wet weights consisting of a composite of soil particles and environmental moisture. In wet transects, collection of a boot sock sample inevitably captures a substantial amount of water from puddles and moist soil. While this water content contributes to differential wet weights, it is absent from TSS dry weights. Additionally, loss of boot sock fabric during sampling may play some role in obtaining negative (non-meaningful) differential wet weights, but this loss of fabric would not affect TSS measurements.
Accounting for these uncertainties in real world scenarios is challenging. Our study has indicated that differential wet weights are likely an overestimate of mass, whereas TSS dry weights may underestimate true sample weight. The choice of approach necessarily impacts *E. coli* concentrations when samples are normalized by weight. As an example, a single boot sock sample weighed 3.12 grams via the differential weight method, but only 0.15 grams when measured using the TSS method. When these weights were used to normalise an *E. coli* concentration of 1.7×10^5 MPN, the same boot sock yielded *E. coli* measurements of 4 log and 6 log MPN per gram of soil using the differential and TSS methods, respectively.
This difficulty in normalization approach highlights the need to consider specific contextual factors when choosing an appropriate method. Overall, our findings suggest that in Fijian informal settlement soils, which typically represented more moist conditions, wet weights may underestimate *E. coli* levels to some extent. However, wet weights are likely preferred given that a high proportion of mass data was meaningful and that laboratory analysts may be spared the additional burden of TSS analysis. However, in conditions where soil is dry or in paved areas, as was often seen in Indonesian communities, the use of the adapted TSS method overcomes the potential difficulties in obtaining meaningful mass data and may be optimal. Thus, the TSS method may be particularly useful for future boot sock studies in arid regions. Further, the successful application of the TSS method in measuring soil weights showcases the method’s versatility in

sampling environmental surfaces in dry or constructed areas. However, whichever normalization approach chosen, it should be understood that possible over- or under-estimation of *E. coli* levels may occur.

The RISE consortium

Member	Affiliation at time of participation	Country
Co-Directors		
Prof. Karin Leder	Monash University	Australia
Prof. Diego Ramirez-Lovering	Monash University	Australia
Chief Investigators		
Prof. Karin Leder	Monash University	Australia
Prof. Diego Ramirez-Lovering	Monash University	Australia
Prof. Ansariadi	Hasanuddin University	Indonesia
Dr Fiona Barker	Monash University	Australia
Prof. Rebekah Brown	Monash University	Australia
Kerrie Burge	Monash University	Australia
Prof. Steven Chown	Monash University	Australia
Prof. Thomas Clasen	Emory University	USA
Adj. Prof Mohamed El Sioufi	Monash University	Australia

	of Technology	
Prof. Julie Simpson	University of Melbourne	Australia
Dr Sheela Sinharoy	Emory University	USA
Ruzka Taruc	RISE	Australia
Autiko Tela	RISE / Fiji National University	Fiji
Dr Amelia Turagabeci	Fiji National University	Fiji
Dr Brandon Winfrey	Monash University	Australia
Prof. Tony Wong	Monash University	Australia

Researchers

Leah Barrett	Monash University	Australia
Dr Lamiya Bata	Monash University	Australia
Dr Dieter Bulach	Melbourne University	Australia
Dr Yussi Delgado	Monash University	Australia
Dr Dewa AP Rasmika Dewi	Monash University	Australia
Genie Fleming	Monash University	Australia
Dr Audrie Lin	University of California, Santa Cruz	USA
Dr Fiona Lynch	Monash University	Australia
Dr John Openshaw	University of Vermont	USA
Dr Joelle Rosser	Stanford University	USA

rise

Powered by Google Translate

REVITALISING INFORMAL SETTLEMENTS AND THEIR ENVIRONMENTS

Sean Mulcair	Monash University	Australia
Natasha Sertori	Monash University	Australia
Maily Tran	Monash University	Australia

Program Management (Monash University)

Mike Gilbert	Monash University	Australia
Zakiya Gora-Ivanoff	Monash University	Australia
Jayden Holmes	Monash University	Australia
Soph Iem	Monash University	Australia
Josphin Johnson	Monash University	Australia
Brietta Kingsley-Miller	Monash University	Australia
Candice Lever	Monash University	Australia
Ashley Peng	Monash University	Australia

Program Delivery (RISE Fiji)

Jonati Kitekitoga Bale	RISE Fiji	Fiji
Salesi Buadromo	RISE Fiji	Fiji
Jannifer Filipe	RISE Fiji	Fiji
Jonathan Holmes	RISE Fiji	Fiji

rise

Powered by Google Translate

REVITALISING INFORMAL SETTLEMENTS AND THEIR ENVIRONMENTS

Mere Naulumatua	RISE Fiji	Fiji
Savu Nofoimuli	RISE Fiji	Fiji
Waisale Rakusa	RISE Fiji	Fiji
Mesuilame Ramacake	RISE Fiji	Fiji
Bulou Ratulevu	RISE Fiji	Fiji
Senirtiki Roqara	RISE Fiji	Fiji
Sereana Seavula	RISE Fiji	Fiji
Meiva Setoka	RISE Fiji	Fiji
Josaiah Thaggard	RISE Fiji	Fiji
Neumi Tuisinu	RISE Fiji	Fiji
Isoa Vakarewa	RISE Fiji	Fiji
Revoni Vamosi	RISE Fiji	Fiji
Meagan Volau	RISE Fiji	Fiji
Taniela Waka	RISE Fiji	Fiji
Mosese Walesi	RISE Fiji	Fiji
Vinaina Waqa	RISE Fiji	Fiji
Alex Wilson	RISE Fiji	Fiji
Iliesa Wise	RISE Fiji	Fiji

[illegible]	[illegible]	[illegible]
Muhammad (Imran) Haerik	RISE Indonesia	Indonesia
Mohammad Hatta	RISE Indonesia	Indonesia
Khaerul Anam Hattas	RISE Indonesia	Indonesia
Herlina	RISE Indonesia	Indonesia
Adrianto Hidayat	RISE Indonesia	Indonesia
Ikram	RISE Indonesia	Indonesia
Noor (Ilham) Ilhamsyah	RISE Indonesia	Indonesia
Nurul Inayah	RISE Indonesia	Indonesia
Triani Puteri Jati	RISE Indonesia	Indonesia
Liza (Icha) Marzaman	RISE Indonesia	Indonesia
Sakka Pati	RISE Indonesia	Indonesia
Nur Intan Putri	RISE Indonesia	Indonesia
Abdul Rajab	RISE Indonesia	Indonesia
Ina Rahlina	RISE Indonesia	Indonesia
Akhnan Rizaldy	RISE Indonesia	Indonesia
Rosnaena	RISE Indonesia	Indonesia
Maghfira Saifuddaolah	RISE Indonesia	Indonesia
Annisa Fildza Shaffira	RISE Indonesia	Indonesia
Supriadi	RISE Indonesia	Indonesia

Fais Achmad	RISE Indonesia	Indonesia
Hajrul Ajwad	RISE Indonesia	Indonesia
Prof. Pascale Allotey	Monash University	Australia
Dian Sidik Arsyad	RISE Indonesia	Indonesia
Asmirayanti	RISE Indonesia	Indonesia
Prof. Stephen Baker	Cambridge University	UK
Ateca Betero	RISE Fiji	Fiji
Daiana Bola	RISE Fiji	Fiji
Bruce Cahan	Stanford University	USA
Amanda Cameron	Monash University	Australia
Ellie Chang	Monash University	Australia
Emily Darlison	Monash University	Australia
Samuela Dau	RISE Fiji	Fiji
Prof. Ana Deletic	Monash University	Australia
Dr Grant Duffy	Monash University	Australia
Indra Dwinata	RISE Indonesia	Indonesia
Dr Moataz Medhat ElQadi	Monash University	Australia
Dr Michelle Escobar-Carias	Monash University	Australia
Dr Peter Faber	Monash University	Australia
Muhammad Faisol	RISE Indonesia	Indonesia

Peter Kolotelo	Monash University	Australia
Apakuki Koroi	RISE Fiji	Fiji
Dr Rachel Lappan	Monash University	Australia
Anna Leersnyder	Monash University	Australia
Dr Robyn Mansfield	Monash University	Australia
Andrew McEvoy	Monash University	Australia
Dr Sarah McGuinness	Monash University	Australia
Dr Mahsa Mesgar	Monash University	Australia
Sailosi Nasaroa	RISE Fiji	Fiji
Geminingsih Nastiti	RISE Indonesia	Indonesia
Wikke Novalia	RISE Indonesia	Indonesia
M. Rizaldi Trias Jaya Putra Nurdin	RISE Indonesia	Indonesia
Dr Joanne O'Toole	Monash University	Australia
Dr Hemali Oza	Emory University	USA
Dr Michaela Prescott	Monash University	Australia
Peni Qauqau	RISE Fiji	Fiji
Dr Noelene Quinsey	Monash University	Australia
Dr Emma Ramsay	Monash University	Australia
Elena Randerath	RISE Fiji	Fiji

rise

Powered by Google Translate

REVITALISING INFORMAL SETTLEMENTS AND THEIR ENVIRONMENTS

Javad Shahidinejad	Monash University	Australia
Semisi Siganisucu	RISE Fiji	Fiji
Saifuddin Sirajuddin	RISE Indonesia	Indonesia
Savitri Soegijoko	RISE Indonesia	Indonesia
Dr Dasha Spasojevic	Monash University	Australia
Uhwan Subhan	RISE Indonesia	Indonesia
Satria Suryahadi	RISE Indonesia	Indonesia
Dr Rohan Sweeney	Monash University	Australia
Pita Tamani	RISE Fiji	Fiji
Bruce Temo	RISE Fiji	Fiji
Daniela Tinios	Monash University	Australia
Jaya Tulha	RISE Indonesia	Indonesia
Dr Christian Urich	Monash University	Australia
Silivia Vilsoni	RISE Fiji	Fiji
Filise Volavola	RISE Fiji	Fiji
Jane Wardani	Monash University	Australia
A/Prof. Eberhard Weber	University of the South Pacific (Fiji)	Fiji
Dr Erich Wolff	Monash University	Australia
Amalie Wright	Monash University	Australia

rise

Powered by Google Translate

REVITALISING INFORMAL SETTLEMENTS AND THEIR ENVIRONMENTS

About People Governance Publications Videos Annual reports News

Get involved

About | Our Approach | Water Sensitive Cities | Program Partners | News | Resources | Get Involved
